# Differences in gait kinematics and spatiotemporal parameters among different Big Five personality types under emotional states: a three-dimensional motion capture study

**DOI:** 10.3389/fpsyg.2026.1801869

**Published:** 2026-05-21

**Authors:** Xiya Guo, Xinyue Cao, Zhengyang He, Xiaoyu Wang

**Affiliations:** College of Design and Arts, Shaanxi University of Science and Technology, Xi'an, China

**Keywords:** Big Five personality, emotion, gait, personality, three-dimensional motion capture

## Abstract

This study addresses the lack of research on the mechanisms linking personality traits, emotional states, and gait patterns by systematically investigating the interactive effects of the Big Five personality traits (neuroticism, extraversion, openness, agreeableness, and conscientiousness) and three emotional states (pleasure, anger, and sadness) on walking gait. Using the Qualisys Track Manager three-dimensional motion capture system, lower limb joint kinematic data and spatiotemporal gait parameters were collected from 30 participants with typical personality profiles (prescreened using standardized scales). The results showed that under all three emotional conditions, individuals high in neuroticism, extraversion, and openness exhibited significantly lower dynamic stability, whereas those high in agreeableness and conscientiousness demonstrated greater stability. Gait speed and locomotor efficiency were significantly reduced in individuals high in neuroticism, agreeableness, and conscientiousness, while those high in extraversion and openness maintained a moderately fast walking speed. Moreover, distinct patterns of joint functional status were observed across different personality types. These findings not only provide quantifiable biomechanical biomarkers for mental health risk assessment but also establish an empirical foundation for personality-specific gait simulation in virtual character modeling.

## 1 Introduction

Gait, as a core indicator of fundamental human motor function, exhibits parameter changes with significant behavioral implications. Traditional theories suggest that even when emotional stimuli are task-irrelevant, high-arousal emotions can still trigger valence-congruent spontaneous postural adjustments (Stins and Beek, 2011; Bouman et al., 2015). However, recent empirical studies have noted that the influence of emotion on behavior requires goal-relevance modulation: only when emotional stimuli are associated with an individual's current task goals do they elicit significant behavioral responses (Calbi et al., 2022; Mancini et al., 2020, 2022; Mirabella, 2018; Mirabella et al., 2023). In contrast, even when emotional stimuli are task-irrelevant, they may still affect central activity through neural mechanisms (Mirabella and Montalti, 2025; Mirabella et al., 2024). Current research frontiers have extended to the regulatory mechanisms of positive/negative emotions and typical emotions (e.g., pleasure, anger) on spatiotemporal gait parameters. Multiple lines of evidence indicate that emotional states can alter muscle activation patterns and gait characteristics via neural pathways (Mahfoudi et al., 2024; Adlou et al., 2025; Homagain and Ehgoetz Martens, 2023; Alvarez et al., 2025; Wu et al., 2024; Xu et al., 2024). Nevertheless, empirical research on the effects of typical emotions on lower limb joint kinematics remains insufficient. Furthermore, studies on the interaction between personality traits and emotions have revealed that individuals high in extraversion exhibit significant increases in gait speed and step length under pleasure conditions, whereas individuals high in openness display richer gait variability patterns in emotionally evocative contexts (Stephan et al., 2018; Satchell et al., 2017). These findings provide a key basis for establishing a three-dimensional personality–emotion–gait interaction model.

However, several critical issues remain to be clarified. Specifically, it remains unknown whether, under emotionally induced conditions, individuals with different levels of the Big Five personality dimensions (neuroticism, extraversion, openness, agreeableness, and conscientiousness) exhibit statistically significant differences in spatiotemporal gait parameters and three-dimensional kinematic characteristics of the hip, knee, and ankle joints. Furthermore, it is unclear whether personality traits exert a moderating effect on emotion-driven gait parameters, and if so, how the underlying physiological and neural mechanisms, along with locomotor efficiency control, mediate this process. To address these questions, the present study conducted a standardized laboratory walking experiment with systematic induction of three emotional states—pleasure, anger, and sadness—to empirically examine the moderating effect of personality-emotion interactions on gait patterns and to quantitatively analyze the gait characteristics of different personality types under specific emotional states, as well as the biomechanical mechanisms underlying them. The findings are expected to provide objective biomechanical indicators for personalized psychological risk assessment and to establish a data-driven foundation for personality-driven gait modeling in virtual reality, game character design, and animation.

## 2 Methods

### 2.1 Participants

To ensure pronounced trait characteristics across personality dimensions and clear between-group differentiation, 100 candidates were recruited in advance. All candidates completed the Big Five Personality Inventory, and individual scores for each personality dimension were calculated using the standardized scoring method. The overall arithmetic mean for each dimension was then computed and used as the core screening threshold. Candidates with scores above the corresponding dimensional mean were retained, while those with scores equal to or below the mean were excluded. Within this pool, candidates were ranked in descending order of scores for each dimension, and the top six individuals per dimension were selected, resulting in a total of 30 participants (17 males, 13 females) across the five personality dimensions, with balanced sample sizes. In cases of tied scores that exceeded the quota of six, or borderline scores only slightly above the mean, priority was given to individuals with higher scores and more prominent subscale ratings on the respective personality dimension, thereby excluding ambiguous cases. The final sample consisted of participants aged 18–25 years, with a height of 173 ± 15 cm. All participants were physically and mentally healthy, had a normal body mass index, had no limb movement disorders, and volunteered to participate by signing informed consent forms. Within 24 h prior to the experiment, none of the participants engaged in strenuous exercise, alcohol consumption, or late-night activities. All participants received prior training to familiarize themselves with the experimental procedure and exhibited normal walking function.

### 2.2 Research method

In this study, an infrared optical motion capture system was used for gait data acquisition. The core acquisition equipment and accompanying analysis software feature high precision and high stability, providing a solid foundation for accurate kinematic data collection and standardized analysis. The Qualisys Track Manager (QTM) system employs infrared optical tracking technology, which is minimally affected by external interference factors such as ambient lighting and skin reflections. Its well-established marker recognition algorithm ensures outstanding overall data acquisition stability. Moreover, the system offers both high spatial resolution and high temporal sampling rate, enabling precise capture of subtle kinematic changes in key lower limb joints such as the hip, knee, and ankle. It supports customized marker placement schemes for gait analysis and allows simultaneous accurate acquisition of multi-dimensional kinematic data, including joint angles and displacements. The accompanying Visual 3D analysis software integrates several professional and adaptable gait analysis templates, with comprehensive post-processing and error correction functions. It exhibits strong statistical compatibility for various types of kinematic data and meets the requirements of gait data processing in multiple scenarios, enabling efficient analysis and standardized processing of gait data. The synergy between the QTM system and Visual 3D software provides reliable methodological support for data acquisition, subsequent processing, and result analysis in this study.

The emotional video stimuli used in this study were independently collected through online platforms. First, keywords such as “pleasure,” “anger,” and “sadness” were used to retrieve video clips. Clips that appeared repeatedly across different platforms and conveyed clear emotional expressions were prioritized to construct an initial stimulus library. Subsequently, all candidate videos were uniformly screened based on duration, image clarity, and audio stability. Clips with image jitter, audio distortion, or durations that did not meet the requirements were excluded, and video clips lasting 2–5 min were retained. The remaining clips were then categorized by emotional semantics, and those with ambiguous emotional orientation were further excluded. On this basis, the final selection of videos was conducted through an emotional induction efficacy validation experiment. Eighty participants were recruited, and changes in their emotional rating scale scores before and after viewing were compared. Paired-sample *t*-tests were used for statistical analysis to identify videos that significantly induced the target emotions. Ultimately, five videos per emotion were selected to form the formal experimental stimulus library. In addition, neutral videos were included as emotional buffer materials. All videos were presented in a randomized order.

First, the laboratory environment was calibrated according to the experimental requirements, and the data acquisition system QTM (Qualisys Track Manager 2021.2, Qualisys, Sweden) was debugged. Second, the experimenter assisted the participants in wearing the experimental equipment and attaching reflective markers and four tracking plates. Third, the participants completed an emotional rating scale and, upon receiving the walking instruction, walked normally on a designated ground track. This process served to collect gait data under a neutral (M0) emotional state while also familiarizing the participants with the experimental task. Subsequently, the participants watched an emotion-inducing video lasting 2–5 min, immediately completed the SAM emotional rating scale to confirm effective emotional induction, and then performed the emotional walking task according to instructions. Finally, the above steps were repeated for each of the three emotional videos of pleasure, anger, and sadness presented in random order. A neutral video was inserted between each emotional video to reset the emotional state, ensuring that participants were not affected by the previous emotional video. The video presentation order was randomized to avoid sequence bias. Gait data under each of the three emotional states were collected using the QTM system for each participant. To ensure the accuracy of the experimental results, each participant completed three full trials of walking under each emotional state, and the average value of each parameter was taken for analysis.

### 2.3 Gait feature extraction and data acquisition

In the QTM software, the gait data collected under each emotional state were pre-processed. First, the reflective markers were labeled. Second, misplaced and missing markers were removed or supplemented. Finally, a four-step gait segment was extracted as one cycle for subsequent data processing. After preliminary marker completion, the data files were converted to C3D format. In Visual 3D, an appropriate human skeletal model was established based on the static data for each emotional condition. The averaged dynamic data from three trials under the same condition were then applied to the established skeletal model within the gait cycle as the basis for calculation.

To eliminate differences in skeletal marker positions caused by individual variations in height, body weight, and limb length, and to improve between-group comparability, the gait data were normalized. Specifically, gait parameters (e.g., step length and step width) were normalized by height (*H*), the normalization processing method is shown in Equation 1:


$$\begin{array}{l} X_{norm}=\frac{X_{raw}}{H} \end{array}$$ (1)

Where *X*_*norm*_ is the normalized value, *X*_*raw*_ is the original parameter, and *H* is the participant's height. This method effectively reduces the interference of individual body size differences on the comparison of gait parameters and enhances the comparability of between-group analyses. To remove high-frequency noise and low-frequency errors introduced by equipment vibration and skin micro-movements during data acquisition, a bandpass filter of 1–20 Hz was applied to the data. This filtering approach effectively eliminates invalid noise interference while preserving the relevant signals of gait movement, thereby avoiding the loss of critical data. The computed gait parameters mainly included spatiotemporal gait parameters and kinematic data of the hip, knee, and ankle joints. A single gait cycle was defined as the period from heel strike of one foot to the subsequent heel strike of the same foot. The stance phase was defined as the period from heel strike to toe-off, and the swing phase as the period from toe-off to the next heel strike.

For each participant, the gait data from three trials under each emotional condition were averaged to obtain the final value. The three emotional conditions were pleasure M1, anger M2, and sadness M3, while the neutral condition M0 served only as a reference.

### 2.4 Statistical analysis

Statistical processing and analysis of the data were performed using SPSS version 27.0. With personality type and emotional state as independent variables and various gait parameters as dependent variables, one-way and two-way analyses of variance were conducted to determine the effects of personality, emotional state, and their interaction on gait parameters. All analyses of variance were followed by multiple comparison corrections using the Bonferroni method to strictly control the Type I error rate. Partial eta squared was reported as a measure of effect size to quantify and evaluate the practical significance of each factor's effect. For correlation analysis, Pearson's correlation coefficient was used to assess the degree of linear association between continuous variables. This method was selected based on the results of tests for normality of data distribution; all variables included in the analysis met the normality assumption. For any variables that did not follow a normal distribution, Spearman's rank correlation was employed as an alternative analytical approach. To address the issue of multiple testing, the Bonferroni correction was simultaneously applied to the correlation analyses to avoid false-positive errors resulting from multiple comparisons.

## 3 Result

### 3.1 Significance analysis of personality and emotion on gait

As shown in Table 1, personality significantly affected the spatiotemporal parameters of gait and the kinematic indicators of the lower limb joints. Emotion significantly affected the spatiotemporal parameters of gait and individual kinematic indicators of the knee and hip joints. Furthermore, when walking under emotional states, personality had a statistically significant effect on the kinematic characteristics of gait, including the knee and hip joints.

**Table 1:** Results of variance analysis on factors affecting walking posture.

Gait indicators		Personality		Emotion		Personality-emotion interaction	
		*F*-value	*P*-value	*F*-value	*P*-value	*F*-value	*P*-value
Ankle joint	Maximum dorsiflexion angle	10.51	**0.00** ^*^	2.02	0.12	0.00	5.05
	Maximum varus angle	6.51	**0.00** ^*^	0.45	0.72	0.99	0.29
	Maximum internal rotation angle	15.39	**0.00** ^*^	0.33	0.8	0.20	1.36
	Maximum plantar flexion angle	1.92	0.11	1.44	0.24	0.00	3.54
	Maximum valgus angle	3.19	**0.02** ^*^	0.41	0.75	0.47	0.99
	Maximum external rotation angle	5.57	**0.00** ^*^	0.87	0.46	0.01	2.36
	Sagittal plane ROM	2.36	0.06	0.53	0.66	0.80	0.65
	Coronal plane ROM	6.24	**0.00** ^*^	0.94	0.42	0.00	2.66
	Transverse plane ROM	6.91	**0.00** ^*^	0.77	0.51	0.21	1.34
Knee joint	Maximum extension angle	3.56	**0.01** ^*^	2.42	0.07	0.99	0.29
	Maximum adduction angle	4.63	**0.00** ^*^	0.13	0.94	1.00	0.12
	Maximum internal rotation angle	1.80	0.14	0.03	0.99	0.99	0.27
	Maximum flexion angle	0.60	0.67	4.50	**0.01** ^*^	0.96	0.41
	Maximum abduction angle	3.62	**0.01** ^*^	0.09	0.97	1.00	**0.04** ^*^
	Maximum external rotation angle	4.76	**0.00** ^*^	0.31	0.82	0.99	0.28
	Sagittal plane ROM	3.47	**0.01** ^*^	8.57	**0.00** ^*^	0.85	0.58
	Coronal plane ROM	1.56	0.19	0.28	0.84	0.99	0.26
	Transverse plane ROM	0.69	0.60	0.51	0.68	1.00	0.22
Hip joint	Maximum flexion angle	2.49	**0.05** ^*^	1.75	0.16	1.00	0.07
	Maximum adduction angle	1.65	0.17	0.23	0.88	1.00	0.11
	Maximum internal rotation angle	5.54	**0.00** ^*^	0.30	0.83	1.00	0.07
	Maximum extension angle	6.99	**0.00** ^*^	0.98	0.40	1.00	**0.04** ^*^
	Maximum abduction angle	1.12	0.35	0.32	0.81	1.00	0.09
	Maximum external rotation angle	8.62	**0.00** ^*^	0.35	0.79	1.00	**0.05** ^*^
	Sagittal plane ROM	3.74	**0.01** ^*^	11.77	**0.00** ^*^	1.00	0.20
	Coronal plane ROM	5.29	**0.00** ^*^	1.67	0.18	0.97	0.37
	Transverse plane ROM	6.18	**0.00** ^*^	0.19	0.90	1.00	0.22
Spatiotemporal parameters	Step length	2.69	**0.03** ^*^	26.69	**0.00** ^*^	0.98	0.34
	Step width	5.15	**0.00** ^*^	0.45	0.72	0.21	1.35
	Walking speed	1.49	0.21	51.38	**0.00** ^*^	0.87	0.55
	Left step length	2.60	**0.04** ^*^	26.43	**0.00** ^*^	0.94	0.45
	Right step length	2.75	**0.03** ^*^	21.61	**0.00** ^*^	0.99	0.28
	Left stance duration	1.35	0.26	34.47	**0.00** ^*^	0.74	0.71
	Right stance duration	1.15	0.34	38.37	**0.00** ^*^	0.66	0.79
	Left swing duration	1.47	0.22	6.66	**0.00** ^*^	0.91	0.50
	Right swing duration	4.24	**0.00** ^*^	5.20	**0.00** ^*^	0.65	0.80

The values in bold with ^*^ indicate statistical significance.

### 3.2 Correlation analysis among personality, emotion, and gait

#### 3.2.1 Correlation analysis among personality, pleasant emotion, and gait

As shown in Table 2, during walking under pleasant emotional conditions, neuroticism and conscientiousness were positively correlated with gait stability and negatively correlated with gait fluency; extraversion, openness, and agreeableness were positively correlated with gait fluency and negatively correlated with gait stability and joint stiffness.

**Table 2:** Correlation analysis among personality, pleasant emotion, and gait.

Motion feature	Neuroticism	Extraversion	Openness	Agreeableness	Conscientiousness
Transverse plane ROM	−0.06	−0.18	−0.08	−0.12	−0.35
Coronal plane ROM	0.06	−0.14	−0.30	−0.18	−0.21
Sagittal plane ROM	0.16	−0.06	0.15	0.10	−0.06
Maximum external rotation angle	−0.06	−0.24	−0.23	0.20	−0.05
Maximum abduction angle	−0.11	0.07	0.13	0.34^*^	0.06
Maximum extension angle	0.21	−0.14	−0.28	0.24	−0.30
Maximum internal rotation angle	−0.09	−0.33	−0.27	0.14	−0.22
Maximum adduction angle	−0.08	−0.06	−0.14	0.24	−0.13
Maximum flexion angle	0.30	−0.17	−0.15	0.29^*^	−0.32
Transverse plane ROM	0.23	−0.09	−0.05	−0.23	0.03
Coronal plane ROM	0.05	0.17	−0.17	−0.14	−0.11
Sagittal plane ROM	0.09	−0.18	−0.18	−0.26	−0.09
Maximum external rotation angle	0.26	0.07	0.18	0.24	0.00
Maximum abduction angle	−0.16	−0.30	−0.01	−0.23	0.04
Maximum flexion angle	−0.20	0.36^*^	0.05	0.16	−0.16
Maximum internal rotation angle	0.43^*^	0.00	0.14	0.06	0.02
Maximum adduction angle	−0.12	−0.17	−0.13	−0.33	−0.04
Maximum extension angle	−0.07	0.09	−0.17	−0.17	−0.23
Transverse plane ROM	−0.04	−0.20	−0.27	−0.25	−0.08
Coronal plane ROM	0.12	−0.08	−0.20	0.07	−0.21
Sagittal plane ROM	0.25	0.01	−0.18	−0.14	−0.02
Maximum external rotation angle	−0.06	0.09	−0.08	0.10	0.07
Maximum valgus angle	−0.19	−0.10	0.04	0.10	0.17
Maximum plantar flexion angle	−0.28	0.04	0.30^*^	0.06	0.09
Maximum internal rotation angle	−0.09	−0.10	−0.31	−0.14	−0.01
Maximum varus angle	−0.11	−0.14	−0.08	0.13	0.04
Maximum dorsiflexion angle	−0.14	0.08	0.26^*^	−0.10	0.12
Right swing duration	−0.12	0.25	0.06	−0.19	−0.15
Left swing duration	0.11	0.14	−0.04	−0.27	−0.20
Right stance duration	0.19	−0.04	0.08	−0.26	−0.33
Left stance duration	0.24	−0.08	0.13	−0.24	−0.38
Right step length	−0.06	−0.05	−0.01	−0.02	0.10
Left step length	−0.09	−0.10	0.19	0.04	0.30
Walking speed	−0.16	−0.08	−0.02	0.20	0.38^*^
Step width	−0.13	0.26	0.06	0.13	0.35^*^
Step length	−0.08	−0.08	0.11	0.01	0.23

#### 3.2.2 Correlation analysis among personality, anger emotion, and gait

As shown in Table 3, when walking under an angry emotional state, neuroticism was positively correlated with gait fluency; extraversion, openness, and agreeableness were positively correlated with joint stiffness; and conscientiousness was positively correlated with gait stability. In addition, neuroticism was negatively correlated with gait stability; extraversion, openness, and agreeableness were negatively correlated with gait symmetry; and conscientiousness was negatively correlated with gait fluency.

**Table 3:** Correlation analysis among personality, anger emotion, and gait.

Motion Feature	Neuroticism	Extraversion	Openness	Agreeableness	Conscientiousness
Transverse plane ROM	−0.07	−0.21	−0.09	−0.01	−0.20
Coronal plane ROM	−0.06	−0.13	−0.35	−0.35	−0.30
Sagittal plane ROM	0.21	0.00	0.09	−0.03	−0.15
Maximum external rotation angle	0.08	−0.02	−0.27	0.23	0.03
Maximum abduction angle	0.11	−0.07	−0.04	0.45^*^	−0.06
Maximum extension angle	0.20	−0.09	−0.23	0.29	−0.27
Maximum internal rotation angle	0.05	−0.12	−0.33	0.23	−0.07
Maximum adduction angle	0.06	−0.17	−0.29	0.20	−0.28
Maximum flexion angle	0.35^*^	−0.09	−0.17	0.27	−0.38
Transverse plane ROM	0.12	−0.11	−0.14	−0.17	0.06
Coronal plane ROM	−0.05	0.01	−0.10	−0.14	−0.07
Sagittal plane ROM	0.18	−0.23	−0.03	−0.16	−0.07
Maximum external rotation angle	0.13	−0.03	0.26^*^	0.15	−0.04
Maximum abduction angle	−0.08	−0.17	−0.07	−0.11	0.10
Maximum flexion angle	−0.26	0.32^*^	−0.01	0.08	0.02
Maximum internal rotation angle	0.21	−0.12	0.10	−0.02	0.02
Maximum adduction angle	−0.12	−0.17	−0.13	−0.33	−0.04
Maximum extension angle	−0.07	0.07	−0.05	−0.11	−0.08
Transverse plane ROM	−0.03	−0.18	−0.29	−0.15	−0.25
Coronal plane ROM	−0.12	0.05	−0.15	0.27	−0.15
Sagittal plane ROM	0.10	−0.15	−0.18	−0.11	−0.18
Maximum external rotation angle	−0.08	0.15	−0.02	0.08	0.08
Maximum valgus angle	−0.33	−0.02	0.03	−0.13	0.24
Maximum plantar flexion angle	−0.13	0.12	0.19	0.01	0.24
Maximum internal rotation angle	−0.09	−0.01	−0.25	−0.06	−0.14
Maximum varus angle	−0.35	0.04	−0.05	0.02	0.14
Maximum dorsiflexion angle	−0.03	−0.04	0.00	−0.11	0.05
Right swing duration	−0.12	0.18	0.22^*^	−0.27	0.14
Left swing duration	−0.02	0.03	0.19	−0.17	−0.03
Right stance duration	0.05	0.03	0.18	−0.20	−0.14
Left stance duration	−0.01	0.06	0.03	−0.31	−0.15
Right step length	0.11	−0.11	−0.11	−0.14	0.06
Left step length	−0.13	0.00	0.10	−0.12	0.14
Walking speed	0.00	−0.07	−0.11	0.11	0.13
Step width	−0.09	0.08	0.04	−0.05	0.36^*^
Step length	−0.02	−0.06	0.00	−0.14	0.10

#### 3.2.3 Correlation analysis among personality, sad emotion, and gait

As shown in Table 4, when walking under a sad emotional state, neuroticism and extraversion were positively correlated with gait fluency and negatively correlated with gait stability; openness and agreeableness were positively correlated with gait stability and negatively correlated with joint stiffness; conscientiousness was positively correlated with gait symmetry and negatively correlated with gait stability.

**Table 4:** Correlation analysis among personality, sad emotion, and gait.

Motion Feature	Neuroticism	Extraversion	Openness	Agreeableness	Conscientiousness
Transverse plane ROM	−0.22	−0.17	−0.24	−0.11	−0.31
Coronal plane ROM	0.16	−0.22	−0.21	−0.14	−0.47
Sagittal plane ROM	0.11	−0.01	−0.06	−0.05	−0.16
Maximum external rotation angle	0.1	−0.05	−0.2	0.23	0.09
Maximum abduction angle	−0.14	0.06	0.01	0.36^*^	0.09
Maximum extension angle	0.32^*^	−0.15	−0.26	0.19	−0.27
Maximum internal rotation angle	0	−0.14	−0.34	0.2	−0.05
Maximum adduction angle	−0.04	−0.1	−0.15	0.31^*^	−0.25
Maximum flexion angle	0.36^*^	−0.14	−0.27	0.15	−0.34
Transverse plane ROM	0.27	−0.19	−0.11	−0.32	−0.1
Coronal plane ROM	0.01	0.14	−0.11	−0.2	−0.11
Sagittal plane ROM	0.07	−0.14	−0.14	−0.1	−0.26
Maximum external rotation angle	0.11	0.09	0.22^*^	0.15	−0.08
Maximum abduction angle	−0.05	−0.27	−0.06	−0.19	0.09
Maximum flexion angle	−0.37	0.39^*^	0	0	0.24^*^
Maximum internal rotation angle	0.29	−0.06	0.11	−0.1	−0.14
Maximum adduction angle	−0.05	−0.19	−0.14	−0.33	0.02
Maximum extension angle	−0.24	0.18	−0.14	−0.1	−0.07
Transverse plane ROM	0	−0.07	−0.13	−0.24	−0.19
Coronal plane ROM	0.08	0	−0.16	0.22	−0.25
Sagittal plane ROM	0.17	−0.1	−0.1	−0.12	−0.3
Maximum external rotation angle	−0.01	0.03	−0.08	0.26	0.04
Maximum valgus angle	−0.42	−0.13	0.07	−0.01	0.24^*^
Maximum plantar flexion angle	−0.15	0.01	0.16	0.15	0.16
Maximum internal rotation angle	−0.01	−0.04	−0.2	0.01	−0.15
Maximum varus angle	−0.3	−0.11	−0.04	0.12	0.05
Maximum dorsiflexion angle	0.04	−0.13	0.07	0.02	−0.23
Right swing duration	−0.41	0.34	0.05	−0.07	0.12
Left swing duration	−0.31	0.3	−0.04	−0.24	0.14
Right stance duration	−0.03	0.25	0.07	0.07	−0.15
Left stance duration	0.15	0.12	0.02	0.03	−0.24
Right step length	−0.13	−0.18	0	−0.2	0.05
Left step length	−0.14	−0.1	0.03	−0.27	0.08
Walking speed	−0.02	−0.27	−0.02	−0.16	0.09
Step width	0.01	−0.01	0.01	−0.18	0.25^*^
Step length	−0.14	−0.15	0.02	−0.24	0.07

### 3.3 Effects of personality-emotion interaction on gait characteristics

#### 3.3.1 Effects of personality-emotion interaction on spatiotemporal gait parameters

**Figure 1:**
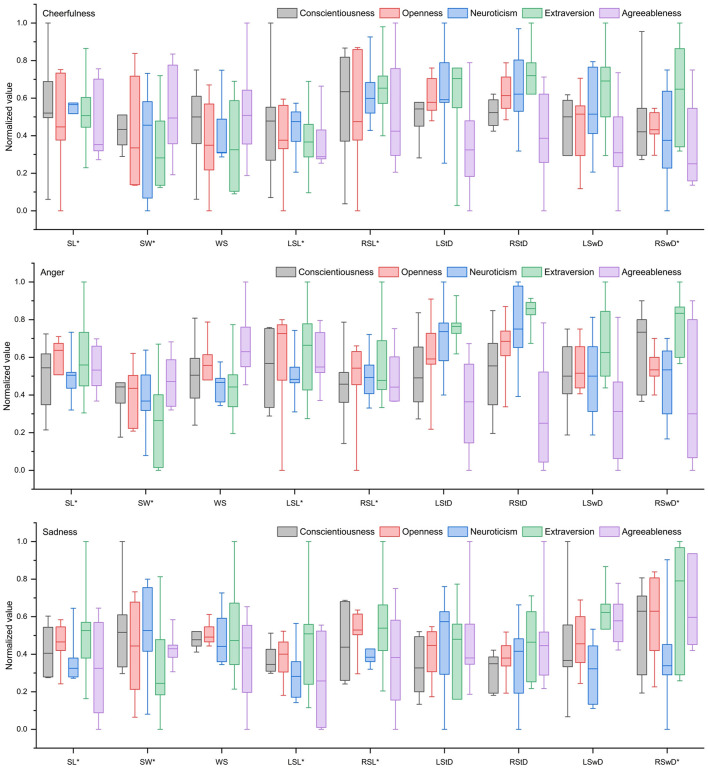
Spatio-temporal parameters of walking gait in three emotional states of different Big Five personality type. *Indicates significant effect.

As shown in Figure 1, when walking under different emotional states, individuals high in neuroticism, extraversion, and agreeableness exhibited different walking speeds, whereas individuals high in conscientiousness maintained normal walking speeds. Individuals high in openness, conscientiousness, and agreeableness showed stable step frequency and step length, while those high in neuroticism displayed greater variability. Individuals high in openness and conscientiousness demonstrated smooth movement continuity. Furthermore, the transition between the swing phase and the stance phase was smoother in individuals high in extraversion and conscientiousness compared to those high in neuroticism.

#### 3.3.2 Effects of personality-emotion interaction on gait kinematic characteristics

**Figure 2:**
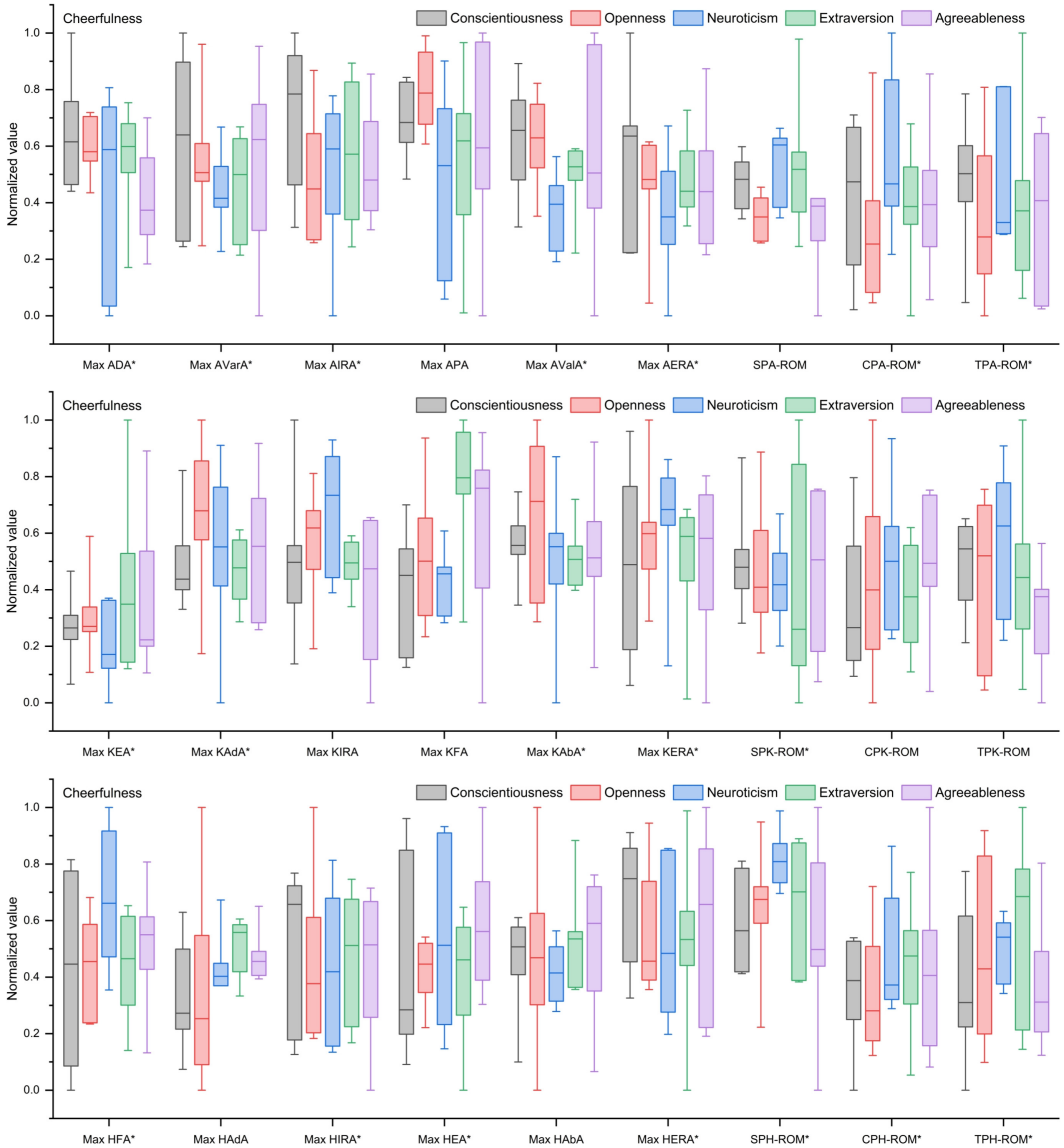
Kinematic characteristics of gait for different Big Five personality types under a pleasant emotional state. *Indicates significant effect.

As shown in Figure 2, under a pleasant emotional state, individuals high in neuroticism and openness tended to exhibit motor control disturbances and postural instability, leading to reduced locomotor efficiency or difficulty in maintaining balance. Individuals high in extraversion displayed overall smooth and efficient gait but weak movement continuity. Individuals high in agreeableness and conscientiousness demonstrated stable and controllable movements with high joint flexibility, emphasizing motor control over speed.

**Figure 3:**
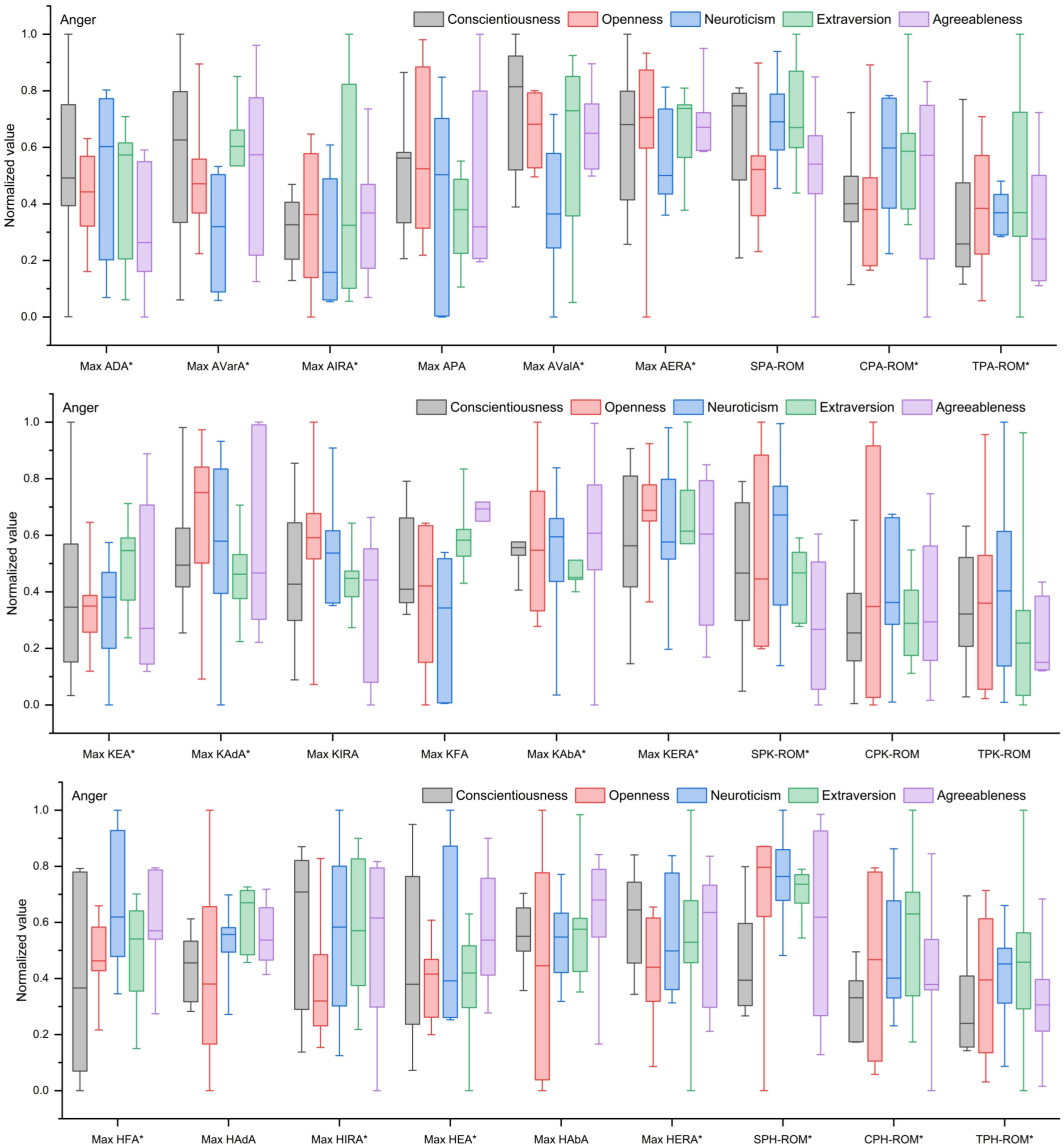
Kinematic characteristics of gait for different Big Five personality types under an angry emotional state. *Indicates significant effect.

As shown in Figure 3, under an angry emotional state, individuals high in neuroticism and agreeableness generally exhibited decreased stability, reduced swing amplitude, poor movement continuity, and low locomotor efficiency. Individuals high in extraversion showed a stable stance phase but reduced swing amplitude and weakened continuity, with enhanced hip lateral movement to improve flexibility. Individuals high in openness demonstrated a stable stance phase and increased swing amplitude, but impaired movement continuity and restricted hip movement. Individuals high in conscientiousness exhibited a stable stance phase and small swing amplitude, resulting in low locomotor efficiency and reduced continuity, along with increased joint movement but poor hip coordination.

**Figure 4:**
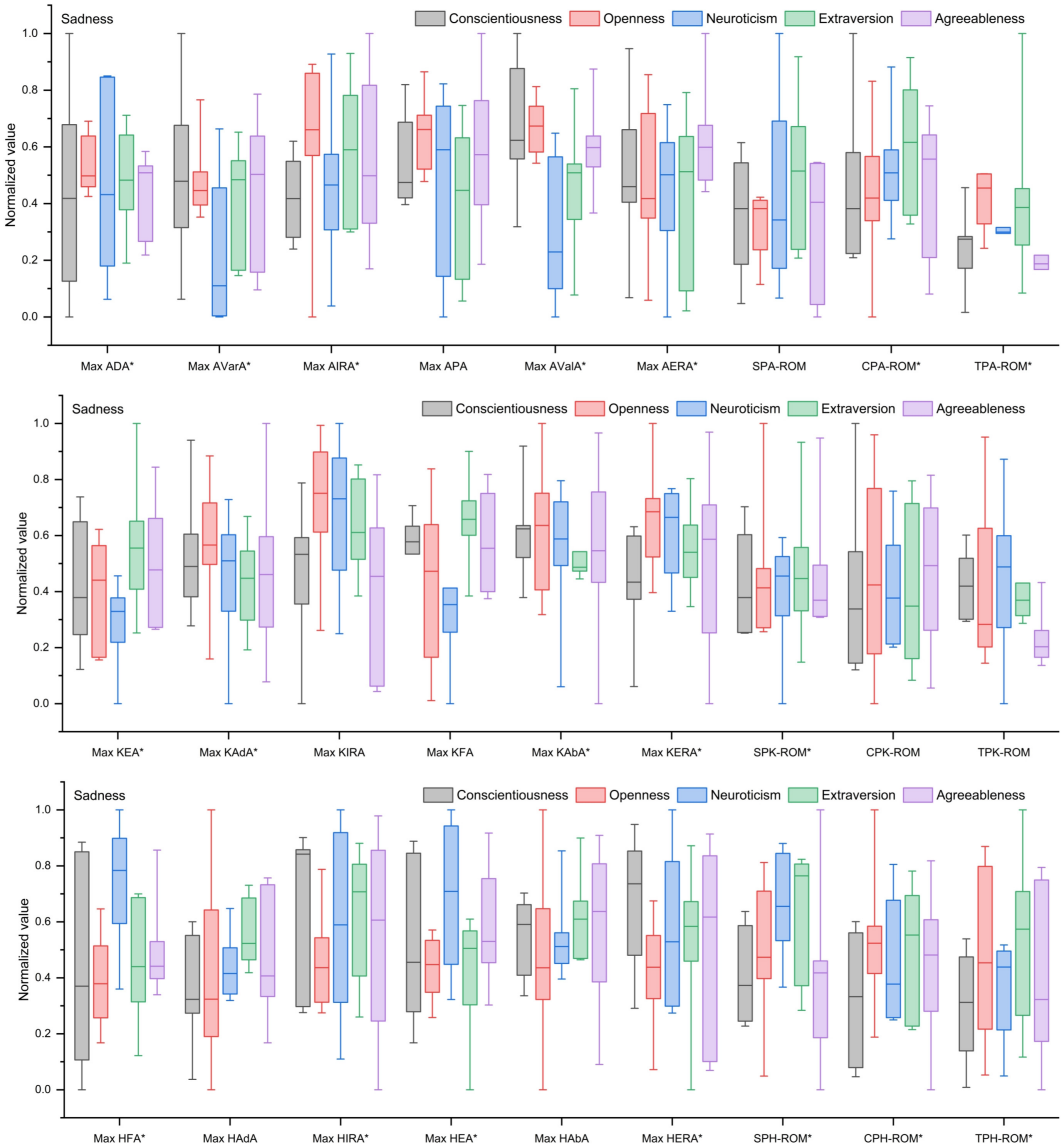
Kinematic characteristics of gait for different Big Five personality types under a sad emotional state. *Indicates significant effect.

As shown in Figure 4, under a sad emotional state, individuals high in neuroticism and conscientiousness generally exhibited unstable stance phase, restricted joint movement, reduced swing amplitude, poor movement continuity, and gait incoordination. Individuals high in extraversion showed a stable stance phase, flexible multi-directional movement, and increased swing amplitude, but movement continuity fluctuated due to weak knee abduction, presenting an overall pattern of dynamic expansion. Individuals high in openness demonstrated a stable stance phase and flexible joint movement, but incoordination between movement continuity and swing amplitude led to restricted gait dynamics. Individuals high in agreeableness exhibited a stable stance phase and enhanced hip abduction with lateral shift, but multi-directional movement was incoordinate and swing amplitude was reduced.

## 4 Discussion

It is evident that the interaction between personality traits and emotional states significantly influences individual gait characteristics. Early research found that regardless of whether emotional stimuli were task-relevant, emotions could automatically induce behavioral responses (Naugle et al., 2010; Stins et al., 2011; Stins and Beek, 2011; Barliya et al., 2013; Bouman et al., 2015), specifically affecting movement speed and fluency (Gross et al., 2012; Kang and Gross, 2015). Among these, a pleasant emotional state typically affects the stance phase of walking (Naugle et al., 2011). These conclusions are consistent with the analysis results of emotion-gait spatiotemporal parameters and joint kinematic features in Table 1 of this paper, thereby validating the regulatory effect of emotion on gait patterns. The underlying mechanism is that individuals, upon receiving emotional stimuli, can automatically regulate postural control through neural pathways, simultaneously enhancing attentional resource allocation to movements and postures themselves. However, recent research on the relationship between emotion and motor behavior presents different theoretical perspectives. Some scholars argue that the influence of emotion on motor behavior is not entirely automatic but is regulated by an individual's current task goals. In other words, emotional stimuli are preferentially processed and consume cognitive resources, thereby affecting motor behavior, only when they are related to task goals (Calbi et al., 2022; Mancini et al., 2020, 2022; Mirabella, 2018; Mirabella et al., 2023). At the same time, even if emotional stimuli are unrelated to task goals, they may still influence brain activity through neural mechanisms, although they may not directly manifest as overt changes in motor responses (Mirabella and Montalti, 2025; Mirabella et al., 2024). Other research indicates that emotions significantly influence motor behavior because of the close interaction between emotion and motor systems at the neurophysiological level, and this influence is even unrelated to task goals (Adlou et al., 2025; Homagain and Ehgoetz Martens, 2023; Alvarez et al., 2025). The experimental task design of this study is consistent with this: participants only needed to complete walking tasks without additional cognitive load. The results show that different emotional stimuli can still cause significant changes in gait characteristics, supporting the view that emotions can directly regulate motor behavior in non-task-related contexts. More importantly, this study further reveals the gait differences among individuals with different personality traits under emotional states. If, according to some previous views, emotion does not affect motor behavior in non-task-related situations, then the emotion-gait variations observed in this study can primarily be attributed to the moderating effect of personality traits. This inference is also consistent with previous research (Stephan et al., 2018; Satchell et al., 2017). Specifically, individuals high in neuroticism often exhibit reduced cognitive function, leading to poorer gait coordination, weaker gait stability, slower walking speed, and lower locomotor efficiency (Luchetti et al., 2016); especially under an angry emotional state, their joint stiffness significantly increases, while under pleasant and sad emotional states, they exhibit moderate stiffness, easily leading to physiological dysregulation (Stephan et al., 2016), thereby directly affecting walking speed. In contrast, individuals high in extraversion have faster information processing speed, which helps achieve more efficient motor control (Pearman, 2009); individuals high in openness have good cognitive function, which helps maintain gait coordination and faster walking speed (Luchetti et al., 2016). Therefore, under the three emotional states, individuals high in extraversion exhibited faster walking speed, high locomotor efficiency, and low joint stiffness; individuals high in openness maintained moderately fast walking speeds and lower knee and ankle joint stiffness. However, it is worth noting that their dynamic stability is relatively weak, which may stem from a compromise in postural control to achieve fast and efficient gait. In contrast, individuals high in agreeableness and conscientiousness also possess superior information processing speed and cognitive function (Luchetti et al., 2016; Pearman, 2009), and their physiological function is not easily imbalanced (Richardson et al., 2015; Rosso et al., 2015; Stephan et al., 2016). Thus, their dynamic stability was consistently strong, especially the high joint flexibility of individuals high in agreeableness. However, their walking speed was slower, locomotor efficiency was lower, and individuals high in conscientiousness had relatively stiff joints. This phenomenon may be due to insufficient differentiation of personality traits among participants in the experiment, leading to some expected behavioral patterns not being fully manifested. In summary, the results of this study indicate that the relationship between emotion and gait is not only regulated by task context but also involves complex interactions with stable individual personality traits. Personality traits jointly shape gait performance under emotional states by influencing cognitive resource allocation, motor control efficiency, and physiological regulatory mechanisms.

The regulatory mechanism of the interaction between personality traits and emotional states on gait parameters explored in this study provides objective evidence for assessing the risk of depression onset and identifying high-risk populations based on biomechanical markers. Previous studies have confirmed that slowed walking speed is a potential risk marker for depression onset in adults, and its predictive validity is not affected by confounding factors such as gender and age (Guimarães et al., 2025; Wang et al., 2021; Chan et al., 2020). Concurrently, specific personality traits such as low extraversion, high neuroticism, and low conscientiousness have been identified as prospective predictors of the development of depressive symptoms (Hakulinen et al., 2015). The underlying mechanisms can be explained from multiple perspectives: at the neurobiological level, gait regulation relies on the coordinated functioning of the dopaminergic system and the prefrontal cortex, and the pathological processes of depression are closely associated with functional abnormalities in these brain regions. Depression-related impairments in synaptic plasticity may weaken neuromotor recruitment, leading to slowed walking speed and abnormal movement patterns (Guimarães et al., 2025; Takakusaki, 2017; Walther et al., 2019; Appelbaum et al., 2023); at the cognitive level, efficient gait control requires the involvement of higher-order cognitive resources such as working memory and executive function, and the cognitive decline associated with depression directly impairs gait coordination and speed (Handing et al., 2023; Hoogendijk et al., 2020); at the behavioral-psychological cycle level, limitations in daily activities caused by gait abnormalities can easily trigger a vicious cycle of “reduced activity → functional decline → decreased social participation → worsening mood,” which continuously exacerbates susceptibility to depression (Stubbs et al., 2016). Notably, the psychological mechanisms underlying core personality traits further reinforce these pathways: individuals with low extraversion exhibit blunted reward responses and weak social support; those with high neuroticism demonstrate heightened sensitivity to negative emotions; and those with low conscientiousness lack the ability to maintain goals and solve problems. Together, these factors constitute adaptive deficits in coping with stress (Hakulinen et al., 2015). Therefore, screening for personality traits using standardized tools such as the Big Five Personality Inventory, while simultaneously collecting gait biomechanical data, enables an objective quantitative assessment of the risk of developing depression.

## 5 Conclusion

Through biomechanical analysis, this study confirms that differences in Big Five personality traits significantly modulate lower limb gait patterns under emotional states, systematically quantifying kinematic indicators of the ankle, knee, and hip joints as well as spatiotemporal gait parameters. These objective findings not only provide quantifiable biomechanical evidence for mental health risk assessment but also establish an empirical foundation for virtual character modeling. Nevertheless, several limitations exist in this study. The narrow age range of the sample limits the generalizability of the findings. The proportion of female samples is insufficient, which cannot effectively reflect the characteristics of the female group. This not only reduces the universality and external validity of the conclusion, but also leads to insufficient statistical test power for the influence of personality and emotion on gait under gender stratification. Only three typical emotions, pleasure, anger, and sadness, were selected, which does not fully capture the diversity of emotional dimensions. The study focused on lower limb kinematic characteristics and did not include key indicators such as upper limb coordination patterns or kinetic parameters. Future research should integrate multi-dimensional emotional stimuli and whole-body kinematic and kinetic indicators to further explore the regulatory mechanisms of personality-emotion interactions on motor behavior, thereby providing a more comprehensive theoretical basis for precise screening of mental health risks and naturalistic modeling of virtual characters.

## Data Availability

The original contributions presented in the study are included in the article/supplementary material, further inquiries can be directed to the corresponding author.
